# Poly[[diaqua-μ_4_-pyrazine-2,3-dicarboxyl­ato-κ^6^ *N*,*O* ^2^:*O* ^2′^:*O* ^3^,*O* ^3′^:*O* ^3^-strontium(II)] monohydrate]

**DOI:** 10.1107/S1600536808015316

**Published:** 2008-06-07

**Authors:** Anita Abedi, Maryam Mousavi Mirkolaei, Vahid Amani

**Affiliations:** a Department of Chemistry, Islamic Azad University, North Tehran Branch, Tehran, Iran; b Department of Chemistry, Islamic Azad University, Shahr-e-Rey Branch, Tehran, Iran

## Abstract

In the title compound, {[Sr(C_6_H_2_N_2_O_4_)(H_2_O)_2_]·H_2_O}_*n*_, the Sr^II^ ions are bridged by the pyrazine-2,3-dicarboxyl­ate ligands with the formation of two-dimensional polymeric layers parallel to the *ac* plane. Each Sr^II^ ion is eight-coordinated by one N and five O atoms from the four ligands and two water mol­ecules. The coordination polyhedron is derived from a  penta­gonal bipyramid with an O atom at the apex on one side of the equatorial plane and two O atoms sharing the apical site on the other side. The coordinated and uncoordinated water mol­ecules are involved in O—H⋯O and O—H⋯N hydrogen bonds, which consolidate the crystal structure.

## Related literature

For related literature, see: Takusagawa & Shimada (1973 ▶); Richard *et al.* (1973 ▶); Zou *et al.* (1999 ▶); Konar *et al.* (2004 ▶); Li *et al.* (2003 ▶); Xu *et al.* (2008 ▶); Ma *et al.* (2006 ▶); Ptasiewicz-Bak & Leciejewicz (1997**a* ▶,b* ▶); Starosta & Leciejewicz (2005 ▶); Tombul *et al.* (2006 ▶).
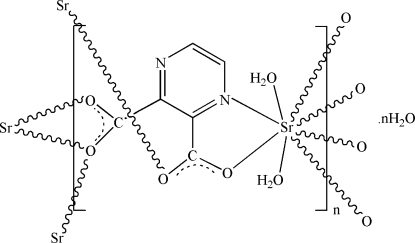


## Experimental

### 

#### Crystal data

[Sr(C_6_H_2_N_2_O_4_)(H_2_O)_2_]·H_2_O
*M* *_r_* = 307.76
Monoclinic, 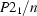

*a* = 10.4931 (7) Å
*b* = 6.9839 (4) Å
*c* = 13.5208 (8) Å
β = 94.2670 (10)°
*V* = 988.10 (10) Å^3^
*Z* = 4
Mo *K*α radiation
μ = 5.48 mm^−1^
*T* = 120 (2) K
0.28 × 0.25 × 0.10 mm

#### Data collection

Bruker SMART 1000 CCD area-detector diffractometer
Absorption correction: multi-scan (*SADABS*; Bruker, 1998 ▶) *T*_min_ = 0.240, *T*_max_ = 0.568
8338 measured reflections
1934 independent reflections
1595 reflections with *I* > 2σ(*I*)
*R*_int_ = 0.040

#### Refinement

*R*[*F* ^2^ > 2σ(*F* ^2^)] = 0.024
*wR*(*F* ^2^) = 0.054
*S* = 1.00
1934 reflections
145 parameters
H-atom parameters constrained
Δρ_max_ = 0.92 e Å^−3^
Δρ_min_ = −0.45 e Å^−3^

### 

Data collection: *SMART* (Bruker, 1998 ▶); cell refinement: *SAINT-Plus* (Bruker, 1998 ▶); data reduction: *SAINT-Plus*; program(s) used to solve structure: *SHELXTL* (Sheldrick, 2008 ▶); program(s) used to refine structure: *SHELXTL*; molecular graphics: *SHELXTL*; software used to prepare material for publication: *SHELXTL*.

## Supplementary Material

Crystal structure: contains datablocks I, global. DOI: 10.1107/S1600536808015316/cv2408sup1.cif

Structure factors: contains datablocks I. DOI: 10.1107/S1600536808015316/cv2408Isup2.hkl

Additional supplementary materials:  crystallographic information; 3D view; checkCIF report

## Figures and Tables

**Table 1:** Selected bond lengths (Å)

Sr1—O2^i^	2.4887 (18)
Sr1—O2*W*	2.5106 (18)
Sr1—O4^ii^	2.5533 (18)
Sr1—O1*W*	2.5937 (19)
Sr1—O3	2.6145 (18)
Sr1—O1^iii^	2.6155 (18)
Sr1—N1	2.714 (2)
Sr1—O2^iii^	2.8517 (18)

Symmetry codes: (i) 
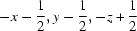
; (ii) 
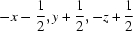
; (iii) 
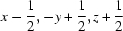
.

**Table 2:** Hydrogen-bond geometry (Å, °)

*D*—H⋯*A*	*D*—H	H⋯*A*	*D*⋯*A*	*D*—H⋯*A*
O1*W*—H1*W*1⋯O3*W*^i^	0.85	1.87	2.713 (3)	170
O1*W*—H2*W*1⋯O3*W*^iv^	0.85	1.90	2.744 (3)	171
O2*W*—H1*W*2⋯O1^ii^	0.85	1.85	2.696 (3)	174
O2*W*—H2*W*2⋯O1*W*^v^	0.85	2.01	2.857 (3)	178
O3*W*—H1*W*3⋯O4	0.85	1.94	2.781 (3)	170
O3*W*—H2*W*3⋯N2^vi^	0.85	1.96	2.792 (3)	168

Symmetry codes: (i) 
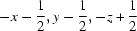
; (ii) 
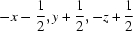
; (iv) 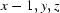; (v) 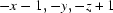; (vi) 
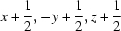
.

**Fig. 1.**
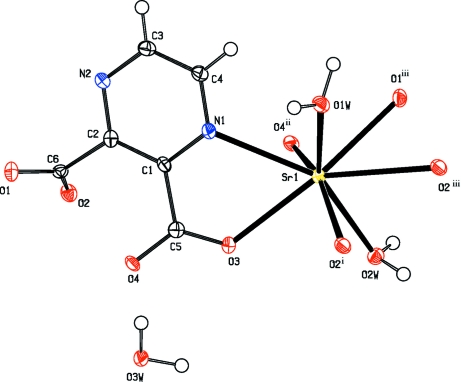
A portion of the polymeric structure of (I) with the atom-numbering scheme and displacement ellipsoids drawn at the 40% probability level [symmetry codes: (i) -x - 1/2,y - 1/2,-z + 1/2, (ii) -x - 1/2, y + 1/2,-z + 1/2, (iii) x - 1/2,-y + 1/2,z + 1/2].

**Fig. 2.**
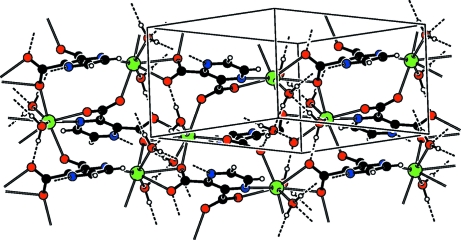
A packing diagram for (I). Hydrogen bonds are shown as dashed lines.

## supplementary crystallographic information

### Comment

Takusagawa & Shimada (1973) first determined the structure of pyrazine-2,3-dicarboxlic acid by single-crystal X-ray analysis. Almost at the same time, the first metal-organic compound of pyrazine-2,3-dicarboxylic acid was reported (Richard *et al.*, 1973). Among many reported compounds containing pyrazine-2,3-dicarboxylic acid, most are complexes of transition metal ions, including manganese (Zou *et al.*, 1999), copper (Konar *et al.*, 2004), zinc (Li *et al.*, 2003), iron (Xu *et al.*, 2008) and cadmium (Ma *et al.*, 2006). Also, there are many reported compounds of pyrazine-2,3-dicarboxylic acid with main group metals such as calcium (Ptasiewicz-Bak & Leciejewicz, 1997*a*; Starosta & Leciejewicz, 2005), magnesium (Ptasiewicz-Bak & Leciejewicz, 1997*b*) and sodium (Tombul *et al.*, 2006) complexes. For further investigation of pyrazine-2,3-dicarboxylic acid, we synthesized the title compound, (I).

The asymmetric unit of the title compound, (Fig. 1), contains molecular sheets in which Sr^II^ ions are bridged by the carboxylate groups of the ligand molecules. Two bridging paths are evident. In the first, an N,*O*-bonding moiety formed by a hetero-ring nitrogen atom and the carboxylate oxygen atom nearest to it and both oxygen atoms of the second carboxylic group are active. The second path is formed by the other oxygen atom from the carboxylic group involved in the N,*O*-bonding moiety and an oxygen atom from the second carboxylic group. The latter atom is bidentate. A two-dimensional molecular pattern is formed. Each Sr^II^ ion is also coordinated by two water oxygen atoms, making the number of coordinated atoms eight. The coordination polyhedron is a distorted pentagonal bipyramid with an oxygen atom at the apex on one side of the equatorial plane and two oxygen atoms forming the apices on the other side. There is also one non-coordinated water molecule in the asymmetric unit. The Sr—O and Sr—N bond lengths are collected in Table 1.

Intermolecular O—H···O and O—H···N hydrogen bonds (Table 2) help to consolidate the crystal packing (Fig. 2).

### Experimental

A solution of pyrazine-2,3-dicarboxlic acid (0.5 g, 2.91 mmol) in methanol (40 ml) was added to a solution of Sr(NO_3_)_2_ (0.31 g, 1.46 mmol) in water (10 ml) and the resulting colourless solution was stirred for 10 min at room temperature. This solution was left to evaporate slowly at room temperature. After one week, colourless plate crystals of the title compound were isolated (yield 0.35 g, 78.03%).

### Refinement

C-bound H atoms were geometrically positioned (C-H 0.95 Å), while O-bound H atoms were found in difference Fourier maps, but placed in idealized positions with O-H of 0.85 Å. All hydrogen atoms were refined in riding model approximation with *U*_iso_(H) = 1.2*U*_eq_ of the paren atom.

### Crystal data

**Table:**

[Sr(C_6_H_2_N_2_O_4_)(H_2_O)_2_]·H_2_O	*F*_000_ = 608
*M**_r_* = 307.76	*D*_x_ = 2.069 Mg m^−^^3^
Monoclinic, *P*2_1_/*n*	Mo *K*α radiation λ = 0.71073 Å
Hall symbol: -P 2yn	Cell parameters from 156 reflections
*a* = 10.4931 (7) Å	θ = 3–26º
*b* = 6.9839 (4) Å	µ = 5.48 mm^−^^1^
*c* = 13.5208 (8) Å	*T* = 120 (2) K
β = 94.2670 (10)º	Plate, colorless
*V* = 988.10 (10) Å^3^	0.28 × 0.25 × 0.10 mm
*Z* = 4	

### Data collection

**Table:**

Bruker SMART 1000 CCD area-detector diffractometer	1934 independent reflections
Radiation source: fine-focus sealed tube	1595 reflections with *I* > 2σ(*I*)
Monochromator: graphite	*R*_int_ = 0.040
*T* = 120(2) K	θ_max_ = 26.0º
φ and ω scans	θ_min_ = 2.4º
Absorption correction: multi-scan(SADABS; Bruker, 1998)	*h* = −12→12
*T*_min_ = 0.240, *T*_max_ = 0.568	*k* = −8→8
8338 measured reflections	*l* = −16→16

### Refinement

**Table:**

Refinement on *F*^2^	Secondary atom site location: difference Fourier map
Least-squares matrix: full	Hydrogen site location: mixed
*R*[*F*^2^ > 2σ(*F*^2^)] = 0.024	H-atom parameters constrained
*wR*(*F*^2^) = 0.054	*w* = 1/[σ^2^(*F*_o_^2^) + (0.026*P*)^2^] where *P* = (*F*_o_^2^ + 2*F*_c_^2^)/3
*S* = 1.01	(Δ/σ)_max_ = 0.002
1934 reflections	Δρ_max_ = 0.92 e Å^−^^3^
145 parameters	Δρ_min_ = −0.45 e Å^−^^3^
Primary atom site location: structure-invariant direct methods	Extinction correction: none

### Special details

**Table:**

Geometry. All e.s.d.'s (except the e.s.d. in the dihedral angle between two l.s. planes) are estimated using the full covariance matrix. The cell e.s.d.'s are taken into account individually in the estimation of e.s.d.'s in distances, angles and torsion angles; correlations between e.s.d.'s in cell parameters are only used when they are defined by crystal symmetry. An approximate (isotropic) treatment of cell e.s.d.'s is used for estimating e.s.d.'s involving l.s. planes.
Refinement. Refinement of *F*^2^ against ALL reflections. The weighted *R*-factor *wR* and goodness of fit *S* are based on *F*^2^, conventional *R*-factors *R* are based on *F*, with *F* set to zero for negative *F*^2^. The threshold expression of *F*^2^ > σ(*F*^2^) is used only for calculating *R*-factors(gt) *etc*. and is not relevant to the choice of reflections for refinement. *R*-factors based on *F*^2^ are statistically about twice as large as those based on *F*, and *R*- factors based on ALL data will be even larger.

### Fractional atomic coordinates and isotropic or equivalent isotropic displacement parameters (Å^2^)

**Table:**

	*x*	*y*	*z*	*U*_iso_*/*U*_eq_	
Sr1	−0.44206 (2)	0.17756 (3)	0.375530 (17)	0.01058 (9)	
N1	−0.4291 (2)	0.1488 (3)	0.17632 (16)	0.0137 (5)	
N2	−0.3958 (2)	0.2550 (3)	−0.01781 (16)	0.0138 (5)	
O1	−0.14140 (18)	0.1344 (3)	−0.07125 (13)	0.0154 (4)	
O2	−0.09707 (18)	0.3655 (3)	0.03862 (13)	0.0148 (4)	
O3	−0.21964 (18)	0.1162 (3)	0.30617 (13)	0.0164 (4)	
O4	−0.10633 (16)	0.0171 (3)	0.18088 (13)	0.0131 (4)	
C1	−0.3115 (2)	0.1518 (4)	0.14221 (19)	0.0114 (6)	
C2	−0.2956 (3)	0.2059 (4)	0.04464 (19)	0.0117 (6)	
C3	−0.5114 (3)	0.2480 (4)	0.0166 (2)	0.0156 (6)	
H3A	−0.5841	0.2794	−0.0264	0.019*	
C4	−0.5277 (3)	0.1963 (4)	0.11335 (19)	0.0150 (6)	
H4A	−0.6115	0.1945	0.1356	0.018*	
C5	−0.2037 (3)	0.0901 (4)	0.21630 (19)	0.0123 (6)	
C6	−0.1670 (3)	0.2340 (4)	0.00239 (19)	0.0114 (6)	
O1W	−0.64324 (17)	−0.0101 (3)	0.30768 (13)	0.0161 (4)	
H1W1	−0.6382	−0.1106	0.2727	0.019*	
H2W1	−0.7174	0.0355	0.2932	0.019*	
O2W	−0.31193 (17)	0.2633 (3)	0.53262 (13)	0.0150 (4)	
H1W2	−0.3220	0.3802	0.5478	0.018*	
H2W2	−0.3246	0.1853	0.5791	0.018*	
O3W	0.11689 (17)	0.1471 (3)	0.28299 (13)	0.0161 (4)	
H1W3	0.0444	0.1203	0.2540	0.019*	
H2W3	0.1011	0.1787	0.3415	0.019*	

### Atomic displacement parameters (Å^2^)

**Table:**

	*U*^11^	*U*^22^	*U*^33^	*U*^12^	*U*^13^	*U*^23^
Sr1	0.00953 (13)	0.01224 (14)	0.01019 (13)	0.00004 (11)	0.00220 (9)	0.00048 (11)
N1	0.0110 (12)	0.0183 (13)	0.0119 (11)	0.0000 (10)	0.0026 (9)	−0.0003 (10)
N2	0.0112 (12)	0.0163 (12)	0.0140 (11)	0.0013 (9)	0.0021 (9)	0.0000 (10)
O1	0.0168 (10)	0.0174 (10)	0.0125 (9)	−0.0015 (8)	0.0050 (8)	−0.0024 (8)
O2	0.0123 (10)	0.0160 (11)	0.0163 (10)	−0.0026 (8)	0.0029 (8)	−0.0017 (8)
O3	0.0142 (10)	0.0245 (11)	0.0106 (9)	0.0028 (8)	0.0022 (8)	−0.0006 (8)
O4	0.0084 (9)	0.0159 (10)	0.0154 (9)	0.0020 (8)	0.0033 (8)	−0.0022 (8)
C1	0.0086 (13)	0.0133 (14)	0.0124 (13)	−0.0008 (11)	0.0016 (10)	−0.0041 (11)
C2	0.0137 (14)	0.0095 (13)	0.0123 (13)	−0.0030 (11)	0.0024 (11)	−0.0016 (11)
C3	0.0119 (15)	0.0185 (14)	0.0161 (14)	0.0013 (11)	−0.0009 (11)	−0.0008 (12)
C4	0.0096 (14)	0.0195 (15)	0.0157 (14)	−0.0006 (11)	0.0005 (11)	−0.0008 (12)
C5	0.0126 (14)	0.0099 (13)	0.0144 (14)	−0.0027 (11)	0.0017 (11)	0.0027 (11)
C6	0.0115 (14)	0.0108 (13)	0.0118 (13)	0.0017 (11)	−0.0001 (11)	0.0027 (11)
O1W	0.0123 (10)	0.0176 (10)	0.0184 (10)	0.0009 (8)	0.0016 (8)	−0.0007 (8)
O2W	0.0161 (10)	0.0145 (10)	0.0142 (10)	−0.0007 (8)	0.0010 (8)	0.0013 (8)
O3W	0.0112 (10)	0.0257 (11)	0.0113 (9)	−0.0009 (8)	0.0012 (8)	−0.0013 (8)

### Geometric parameters (Å, °)

**Table:**

Sr1—O2^i^	2.4887 (18)	O2—Sr1^ii^	2.4887 (18)
Sr1—O2W	2.5106 (18)	O2—Sr1^v^	2.8517 (18)
Sr1—O4^ii^	2.5533 (18)	O3—C5	1.252 (3)
Sr1—O1W	2.5937 (19)	O4—C5	1.267 (3)
Sr1—O3	2.6145 (18)	O4—Sr1^i^	2.5533 (18)
Sr1—O1^iii^	2.6155 (18)	C1—C2	1.394 (4)
Sr1—N1	2.714 (2)	C1—C5	1.517 (4)
Sr1—O2^iii^	2.8517 (18)	C2—C6	1.516 (4)
Sr1—C6^iii^	3.082 (3)	C3—C4	1.381 (4)
Sr1—Sr1^iv^	4.4235 (5)	C3—H3A	0.9500
Sr1—H1W2	2.9292	C4—H4A	0.9500
Sr1—H2W2	2.9320	C6—Sr1^v^	3.082 (3)
N1—C4	1.332 (3)	O1W—H1W1	0.8500
N1—C1	1.349 (3)	O1W—H2W1	0.8500
N2—C3	1.331 (3)	O2W—H1W2	0.8500
N2—C2	1.343 (3)	O2W—H2W2	0.8500
O1—C6	1.260 (3)	O3W—H1W3	0.8501
O1—Sr1^v^	2.6155 (18)	O3W—H2W3	0.8499
O2—C6	1.252 (3)		
			
O2^i^—Sr1—O2W	75.75 (6)	O2^iii^—Sr1—H1W2	70.9
O2^i^—Sr1—O4^ii^	157.35 (6)	C6^iii^—Sr1—H1W2	76.3
O2W—Sr1—O4^ii^	85.61 (6)	Sr1^iv^—Sr1—H1W2	78.0
O2^i^—Sr1—O1W	79.88 (6)	O2^i^—Sr1—H2W2	62.3
O2W—Sr1—O1W	142.83 (6)	O2W—Sr1—H2W2	15.6
O4^ii^—Sr1—O1W	122.57 (6)	O4^ii^—Sr1—H2W2	100.6
O2^i^—Sr1—O3	84.44 (6)	O1W—Sr1—H2W2	128.0
O2W—Sr1—O3	84.19 (6)	O3—Sr1—H2W2	90.9
O4^ii^—Sr1—O3	80.93 (6)	O1^iii^—Sr1—H2W2	91.2
O1W—Sr1—O3	121.00 (6)	N1—Sr1—H2W2	152.2
O2^i^—Sr1—O1^iii^	114.76 (6)	O2^iii^—Sr1—H2W2	60.0
O2W—Sr1—O1^iii^	92.48 (6)	C6^iii^—Sr1—H2W2	76.1
O4^ii^—Sr1—O1^iii^	78.28 (6)	Sr1^iv^—Sr1—H2W2	54.3
O1W—Sr1—O1^iii^	72.81 (6)	H1W2—Sr1—H2W2	28.2
O3—Sr1—O1^iii^	159.14 (6)	C4—N1—C1	117.7 (2)
O2^i^—Sr1—N1	112.29 (6)	C4—N1—Sr1	121.46 (17)
O2W—Sr1—N1	142.46 (6)	C1—N1—Sr1	116.91 (16)
O4^ii^—Sr1—N1	75.33 (6)	C3—N2—C2	117.5 (2)
O1W—Sr1—N1	73.21 (6)	C6—O1—Sr1^v^	99.33 (16)
O3—Sr1—N1	61.32 (6)	C6—O2—Sr1^ii^	153.65 (17)
O1^iii^—Sr1—N1	114.22 (6)	C6—O2—Sr1^v^	88.36 (15)
O2^i^—Sr1—O2^iii^	68.33 (6)	Sr1^ii^—O2—Sr1^v^	111.67 (6)
O2W—Sr1—O2^iii^	71.13 (6)	C5—O3—Sr1	124.02 (17)
O4^ii^—Sr1—O2^iii^	117.81 (5)	C5—O4—Sr1^i^	132.10 (16)
O1W—Sr1—O2^iii^	73.99 (5)	N1—C1—C2	120.3 (2)
O3—Sr1—O2^iii^	146.69 (6)	N1—C1—C5	115.2 (2)
O1^iii^—Sr1—O2^iii^	47.66 (5)	C2—C1—C5	124.4 (2)
N1—Sr1—O2^iii^	146.41 (6)	N2—C2—C1	121.3 (2)
O2^i^—Sr1—C6^iii^	91.29 (7)	N2—C2—C6	114.0 (2)
O2W—Sr1—C6^iii^	82.66 (6)	C1—C2—C6	124.4 (2)
O4^ii^—Sr1—C6^iii^	99.08 (6)	N2—C3—C4	121.4 (3)
O1W—Sr1—C6^iii^	70.19 (6)	N2—C3—H3A	119.3
O3—Sr1—C6^iii^	166.80 (6)	C4—C3—H3A	119.3
O1^iii^—Sr1—C6^iii^	23.79 (6)	N1—C4—C3	121.7 (3)
N1—Sr1—C6^iii^	131.61 (7)	N1—C4—H4A	119.1
O2^iii^—Sr1—C6^iii^	23.97 (6)	C3—C4—H4A	119.1
O2^i^—Sr1—Sr1^iv^	36.81 (4)	O3—C5—O4	126.5 (2)
O2W—Sr1—Sr1^iv^	69.70 (4)	O3—C5—C1	116.9 (2)
O4^ii^—Sr1—Sr1^iv^	145.08 (4)	O4—C5—C1	116.6 (2)
O1W—Sr1—Sr1^iv^	73.93 (4)	O2—C6—O1	124.1 (2)
O3—Sr1—Sr1^iv^	118.96 (4)	O2—C6—C2	117.4 (2)
O1^iii^—Sr1—Sr1^iv^	78.55 (4)	O1—C6—C2	118.3 (2)
N1—Sr1—Sr1^iv^	138.62 (5)	O2—C6—Sr1^v^	67.67 (14)
O2^iii^—Sr1—Sr1^iv^	31.52 (4)	O1—C6—Sr1^v^	56.88 (13)
C6^iii^—Sr1—Sr1^iv^	54.80 (5)	C2—C6—Sr1^v^	167.25 (17)
O2^i^—Sr1—H1W2	90.3	Sr1—O1W—H1W1	122.1
O2W—Sr1—H1W2	15.7	Sr1—O1W—H2W1	126.8
O4^ii^—Sr1—H1W2	72.9	H1W1—O1W—H2W1	105.9
O1W—Sr1—H1W2	144.7	Sr1—O2W—H1W2	111.4
O3—Sr1—H1W2	91.3	Sr1—O2W—H2W2	111.6
O1^iii^—Sr1—H1W2	81.0	H1W2—O2W—H2W2	114.0
N1—Sr1—H1W2	140.8	H1W3—O3W—H2W3	104.9
			
O2^i^—Sr1—N1—C4	121.4 (2)	C3—N2—C2—C1	−0.7 (4)
O2W—Sr1—N1—C4	−143.01 (19)	C3—N2—C2—C6	−175.0 (2)
O4^ii^—Sr1—N1—C4	−81.1 (2)	N1—C1—C2—N2	−0.4 (4)
O1W—Sr1—N1—C4	50.2 (2)	C5—C1—C2—N2	178.5 (2)
O3—Sr1—N1—C4	−168.7 (2)	N1—C1—C2—C6	173.3 (2)
O1^iii^—Sr1—N1—C4	−11.5 (2)	C5—C1—C2—C6	−7.8 (4)
O2^iii^—Sr1—N1—C4	37.4 (3)	C2—N2—C3—C4	1.3 (4)
C6^iii^—Sr1—N1—C4	8.1 (2)	C1—N1—C4—C3	−0.3 (4)
Sr1^iv^—Sr1—N1—C4	89.0 (2)	Sr1—N1—C4—C3	156.5 (2)
O2^i^—Sr1—N1—C1	−81.51 (18)	N2—C3—C4—N1	−0.8 (4)
O2W—Sr1—N1—C1	14.0 (2)	Sr1—O3—C5—O4	−162.37 (19)
O4^ii^—Sr1—N1—C1	75.96 (18)	Sr1—O3—C5—C1	17.6 (3)
O1W—Sr1—N1—C1	−152.72 (19)	Sr1^i^—O4—C5—O3	96.7 (3)
O3—Sr1—N1—C1	−11.61 (17)	Sr1^i^—O4—C5—C1	−83.2 (3)
O1^iii^—Sr1—N1—C1	145.59 (17)	N1—C1—C5—O3	−27.8 (3)
O2^iii^—Sr1—N1—C1	−165.53 (15)	C2—C1—C5—O3	153.3 (3)
C6^iii^—Sr1—N1—C1	165.13 (16)	N1—C1—C5—O4	152.2 (2)
Sr1^iv^—Sr1—N1—C1	−113.97 (17)	C2—C1—C5—O4	−26.8 (4)
O2^i^—Sr1—O3—C5	115.1 (2)	Sr1^ii^—O2—C6—O1	148.1 (3)
O2W—Sr1—O3—C5	−168.7 (2)	Sr1^v^—O2—C6—O1	7.3 (3)
O4^ii^—Sr1—O3—C5	−82.2 (2)	Sr1^ii^—O2—C6—C2	−26.3 (5)
O1W—Sr1—O3—C5	40.4 (2)	Sr1^v^—O2—C6—C2	−167.1 (2)
O1^iii^—Sr1—O3—C5	−87.1 (3)	Sr1^ii^—O2—C6—Sr1^v^	140.8 (4)
N1—Sr1—O3—C5	−4.08 (19)	Sr1^v^—O1—C6—O2	−8.1 (3)
O2^iii^—Sr1—O3—C5	149.62 (18)	Sr1^v^—O1—C6—C2	166.25 (19)
C6^iii^—Sr1—O3—C5	−173.4 (3)	N2—C2—C6—O2	110.2 (3)
Sr1^iv^—Sr1—O3—C5	128.36 (19)	C1—C2—C6—O2	−63.9 (4)
C4—N1—C1—C2	0.9 (4)	N2—C2—C6—O1	−64.5 (3)
Sr1—N1—C1—C2	−157.03 (19)	C1—C2—C6—O1	121.4 (3)
C4—N1—C1—C5	−178.1 (2)	N2—C2—C6—Sr1^v^	−0.2 (9)
Sr1—N1—C1—C5	24.0 (3)	C1—C2—C6—Sr1^v^	−174.3 (7)

Symmetry codes: (i) −*x*−1/2, *y*−1/2, −*z*+1/2; (ii) −*x*−1/2, *y*+1/2, −*z*+1/2; (iii) *x*−1/2, −*y*+1/2, *z*+1/2; (iv) −*x*−1, −*y*, −*z*+1; (v) *x*+1/2, −*y*+1/2, *z*−1/2.

### Hydrogen-bond geometry (Å, °)

**Table:**

*D*—H···*A*	*D*—H	H···*A*	*D*···*A*	*D*—H···*A*
O1W—H1W1···O3W^i^	0.85	1.87	2.713 (3)	170
O1W—H2W1···O3W^vi^	0.85	1.90	2.744 (3)	171
O2W—H1W2···O1^ii^	0.85	1.85	2.696 (3)	174
O2W—H2W2···O1W^iv^	0.85	2.01	2.857 (3)	178
O3W—H1W3···O4	0.85	1.94	2.781 (3)	170
O3W—H2W3···N2^vii^	0.85	1.96	2.792 (3)	168

Symmetry codes: (i) −*x*−1/2, *y*−1/2, −*z*+1/2; (vi) *x*−1, *y*, *z*; (ii) −*x*−1/2, *y*+1/2, −*z*+1/2; (iv) −*x*−1, −*y*, −*z*+1;  (vii) *x*+1/2, −*y*+1/2, *z*+1/2.
